# Numerical Simulation and Experimental Analysis of the Semi-Solid Thixotropic Extrusion Forming Process for Producing the Thin-Wall Wrought Aluminum Alloy Mobile Phone Shells

**DOI:** 10.3390/ma14133505

**Published:** 2021-06-23

**Authors:** Yi Guo, Yongfei Wang, Shengdun Zhao

**Affiliations:** 1 School of Energy and Power Engineering, Xi’an Jiaotong University, Xi’an 710049, China; yiguo666@mail.xjtu.edu.cn; 2 State Key Laboratory of Compressor Technology (Anhui Laboratory of Compressor Technology), Hefei 230031, China; 3 School of Mechanical Engineering, Xi’an Jiaotong University, Xi’an 710049, China; sdzhao@mail.xjtu.edu.cn; 4 State Key Laboratory of Materials Processing and Die & Mould Technology, Huazhong University of Science and Technology, Wuhan 430074, China

**Keywords:** wrought aluminum alloy, mobile phone shell, semi-solid thixotropic extrusion forming, numerical simulation, microstructure

## Abstract

Aluminum alloys have been widely used in various engineering applications due to their excellent physical properties such as low density, high strength and good cutting capacity. In this paper, the semi-solid thixotropic extrusion forming process is proposed to produce aluminum alloy 6063 shells for mobile phones. The effects of the operating parameters on the equivalent stress distribution, velocity field, temperature field, and the load of the top mould were investigated through numerical simulations. Optimal parameters were identified from the simulation results. The experiment was then conducted at these optimal parameters. The macromorphology and microstructure results of the mobile phone shells produced from the experiment are presented and discussed. It was found that the optimal process parameters for preparing aluminum alloy 6063 shell by the semi-solid thixotropic extrusion process were a billet temperature of 630 °C, mould temperature of 400 °C, and top mould speed of 10 mm/s. It was found that the mobile phone shells fabricated under the optimal operating conditions were fully filled with a clear outline and a smooth surface. The solid grains in the microstructure were small, uniform and nearly spherical. The average grain size of the microstructure for the product was obtained as 76.92 μm and the average shape factor was found as 0.76.

## 1. Introduction

Al-Mg-Si-based wrought aluminium alloys are attractive materials applied in the computers, communication, and consumer electronics (3C) and automotive industries because of their excellent mechanical properties. They are usually used to fabricate parts by hot forging, machining, and casting processes. However, some shortcomings are observed during the traditional manufacturing processes, which include high production cost [1], high deformation load, low material usage coefficient, low efficiency [2], gas and shrinkage porosity [3]. These problems can be effectively solved by the semi-solid metal forming (SSMF) process, which integrates the benefits of the easy fluidity of the liquid casting process [4] and the high mechanical properties of the solid-state plastic process [5] and consequently is a popular near-net-shape forming process [6].

The semi-solid thixotropic extrusion forming (SSTEF) process is one of the representative techniques used in SSMF processes, which can be defined as a forming process where semi-solid billets with fine and spherical solid grains are extruded into final parts in a preheated mould [7]. The quality of semi-solid billets has an important influence on the filling process, microstructure and mechanical properties of the SSTEF processed components, which can be fabricated by either solid or liquid state routes. Many processing routes have been developed for preparing the fine and near-spherical microstructures. Xu et al. [8] used strain-induced melt activation (SIMA) to prepare semi-solid billets. The AZ91D magnesium alloy experiencing the deformation of repetitive upsetting-extrusion at 340 °C for three cycles was isothermally treated at 580 °C for 10 min, and then semi-solid billets with an average grain size of 59.87 μm and a shape factor of 0.84 were successfully fabricated, which are suitable for investigating the constitutive behavior of a SIMA-processed magnesium alloy. Wang et al. [9] employed a semi-solid isothermal treatment (SSIT) process to prepare semi-solid billets, and an ideal aluminum 6061 semi-solid microstructure with an average grain size of 48.6 μm and shape factor of 0.76 was obtained. Wang et al. [10] also prepared qualified 6063 aluminum alloy semi-solid billets by a recrystallization and partial melting (RAP) process, in which warm radial forging and SSIT were used. Haghayeghi et al. [11] reported the semi-solid Al-7Si-0.3Mg alloy produced by mechanical stirring, and the results showed that the optimum temperature and time for a semi-solid ally, with 700-rpm shear rate were 590 °C and 20 min. Van et al. [12] studied the effects of pouring temperature, slope length, and the slope temperature in cooling slope casting on the formation of fine and spherical microstructures of Al-7Si-Mg alloy, and they found that finer and more spherical solid grains were fabricated at the pouring temperature of 625 °C, the slope length of 250 mm, and the slope temperature at room temperature. Zheng et al. [13] proposed a direct ultrasonic treatment (DUT) process to prepare semi-solid Sn-62Bi billets, they concluded that the average size of primary Bi blocks of Sn-62Bi alloys decreased considerably from about 100 μm of the conventional alloy without DUT treatment to 30–80 μm after treated by DUT at 140–150 °C.

Among the above routes for preparing the semi-solid billets, SIMA and RAP including deformation and SSIT steps are two of the most widely used methods because of their low processing costs [14] and easy implementation of fabricating semi-solid billet with high solid fraction [15]. Therefore, SIMA and RAP have been investigated by researchers for the SSTEF process. Jiang et al. [16] reported the microstructures and mechanical properties of SSTEF processed satellite angle frame components. It was found that semi-solid AZ91D magnesium billets with the average grain size of 5 um could be prepared by the SIMA process which includes equal channel angular extrusion and SSIT processes. Moreover, both the room temperature and high-temperature mechanical properties of the formed components were enhanced effectively. Chen et al. [17] examined the microstructure and mechanical properties of AM60 magnesium alloy bowl-shaped parts thixoformed from as-cast and accumulative plastic deformation (APD) states. It was concluded that semi-solid AM60 billets with small and uniform grains could be fabricated by using APD in the RAP process. The tensile mechanical properties of the product thixoformed from APD deformed alloys showed a significant advantage over those thixoformed from the as-cast alloys. 

Besides the SIMA and RAP processes, a novel process was proposed in our previous study to fabricate the 6063 aluminum semi-solid billets, which was called “radial forging strain-induced melt activated (RFSIMA)” where the radial forging method is used in the SIMA process [18,19]. The process combined the cold radial forging of 6063 alloys as a deformation step and the subsequent isothermal treatment at 625 to 635 °C as an SSIT step. It was demonstrated that semi-solid 6063 billets with fine, near-spherical solid grains and better thixotropic behavior could be fabricated through the RFSIMA process. Therefore, RFSIMA is applied in the SSTEF process for producing high-quality thin-wall wrought aluminum alloy mobile phone shells in this paper. 

To the best knowledge of the authors, thin-wall wrought aluminum alloy parts manufactured by a SSTEF process which includes RFSIMA and extrusion forming have not been reported. Moreover, little research has been performed on the filling ability of semi-solid 6063 billets and the forming quality of mobile phone shells. In this study, the effects of the operating parameters on the equivalent stress distribution, velocity field, temperature field, and the load of the top mould were studied by numerical simulations. Furthermore, the experiment was conducted based on the optimal parameters identified from the simulation results. The macro morphology and microstructure of SSTEF processed mobile phone shells were examined and discussed.

## 2. Semi-Solid Thixotropic Extrusion Forming Scheme for the Mobile Phone Shell

**Figure 1:**
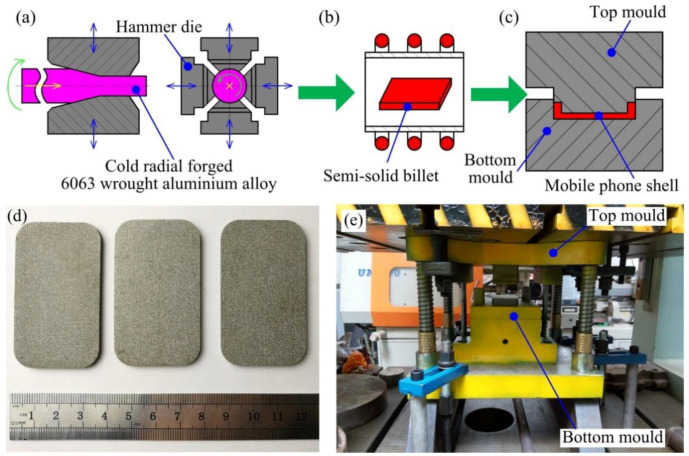
Schematic illustration of the semi-solid thixotropic extrusion forming process for the mobile phone shell: (**a**) Step A—deformation, (**b**) Step B—SSIT, (**c**) Step C—forming, (**d**) Picture of the forming samples, and (**e**) Picture of the forming mould.

The chemical composition of the 6063 alloys used in this work is shown in Table 1. Its solidus and liquidus temperatures are 615 °C, and 655 °C, respectively [18]. The SSTEF process of the thin-wall wrought aluminum alloy mobile phone shell in this work mainly includes three steps, as shown in Figure 1. Step A: The CRF process was applied on the 6063 alloy bar for the deformation from the diameter of 100 to 60 mm (i.e., the area reduction of 64%) [20], and the schematic diagram of the CRF process was shown in Figure 1a. After that, the forming samples as shown in Figure 1d were cut from the deformed alloy along the radial forging direction, which had a length of 60 mm, a width of 35 mm, and a height of 2.5 mm. Step B: These samples are treated by the SSIT process with different billet temperatures and the processing time of 10 min to fabricate the semi-solid billets, and the schematic diagram of the SSIT process was shown in Figure 1b. Step C: The semi-solid samples were thixotropic extruded to obtain the thin-wall wrought aluminum alloy mobile phone shells by the top and bottom moulds at different mould temperatures and different extrusion speeds, the schematic diagram of forming process was shown in Figure 1c, and the picture of forming mould was shown in Figure 1e.

## 3. Simulation Settings of Process Parameters

**Figure 2:**
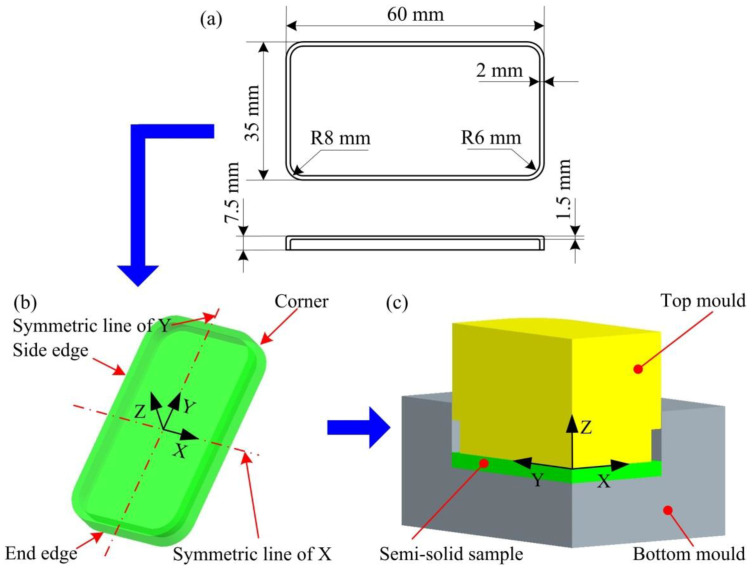
Schematic diagram of thixotropic extrusion forming model for the mobile phone shell: (**a**) dimensions of phone shell, (**b**) three-dimensional model, and (**c**) simulation model.

Figure 2a,b present the dimensions of the thin-wall wrought aluminum alloy mobile phone and the three-dimensional model of this phone shell in this study, respectively. Considering the structural symmetry of the mobile phone shell shown in Figure 2b, 1/4 of the mobile phone shell is calculated in the simulation process. The three-dimensional structure of the simulation model is shown in Figure 2c, mainly including semi-solid samples, top, and bottom moulds. In the simulation, the true stress-true strain curves of the cold radial forged 6063 aluminium alloy obtained from our previous research work [18] are input into the DEFORM-3D (DEFORM-3D V10, SFTC, Columbus, OH, USA) software as the flow stress model of semi-solid material. 

During the SSTEF process, three process parameters including the billet temperature, mold temperature, and extrusion speed, significantly affect the filling ability of semi-solid billet and the forming part quality. To investigate the effect of process parameters, the simulation is conducted by varying the process parameter one by one while keeping the other two parameters invariant. Simulations are classified into four groups as shown in Table 2. 

In Group I, the billet temperature of 630 °C, the mould temperature of 400 °C, and the extrusion speed of 10 mm/s are used to study the filling procedure. The solidification process of the mobile phone shell is investigated by Groups II-IV. In Group II, the billet temperature is set from 625–635 °C with the mould temperature of 400 °C and the extrusion speed of 10 mm/s, while in Group III, the mould temperature varies from 350–450 °C with the billet temperature of 630 °C and the extrusion speed of 10 m/s. In Group IV, the extrusion speed is changed in the range of 5–15 mm/s under the blank and mould temperatures of 630 °C and 400 °C, respectively. 

## 4. Experimental Setup and Microstructure Analysis

The experiment for manufacturing the mobile phone shells was carried out by the SSTEF process under the recommended process parameters determined from the simulation analysis. In the experiment, three mobile phone shells were formed by the SSTEF process to provide samples for the microstructure analysis. It is worthy to note that, the microstructure analysis is only performed on one cross-section of the phone shell in order to show the results of the preliminary optimization based on the simulation results. Further optimization is needed by experimental investigation on the microstructure of different cross-sections of the mobile phone, which would be carried out in our future study. 

**Figure 3:**
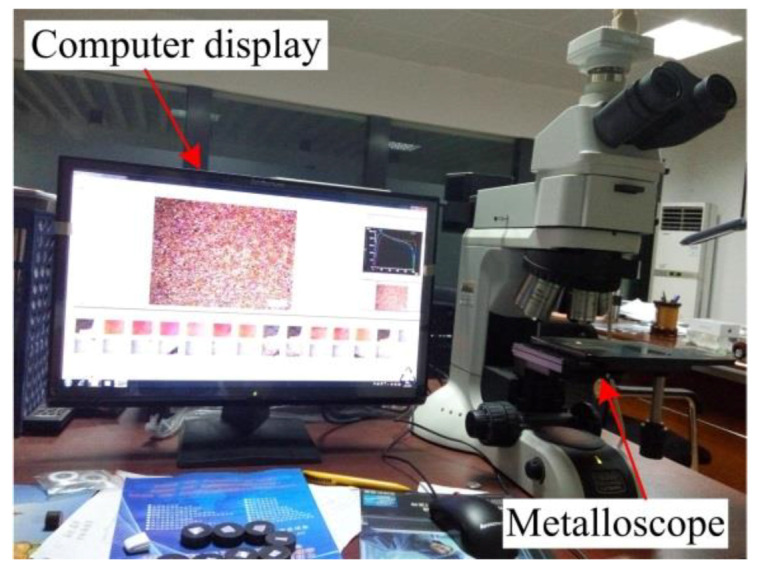
Picture of the optical microscope used in this work.

The quality of the phone shell formed by the SSTEF process is presented by the microstructure analysis of the samples. Samples cutting from different positions of the forming components were ground by the sandpapers with the grits from 200 to 1500, polished via the diamond paste with the particle size of 0.1 μm, and then etched in the aqueous HF solution with a concentration of 5% for about 90 s. Then, the microstructure was observed by the optical microscope (NIKON ECLIPSE LV 150N, Nikon, Tokyo, Japan), which is shown in Figure 3. 

The average grain size (*D*) and the shape factor (*F*) of solid grains can be calculated by Equations (1) and (2), respectively [15,21,22]:

(1) $$D=\frac{\sum_{N=1}^{N}\sqrt{4A/\pi }}{N}$$

(2) $$F=\frac{\sum_{N=1}^{N}4\pi A/P^{2}}{N}$$

where *A*, *N*, *P* are the area, number, perimeter of solid grains, respectively.

## 5. Results and Discussion

### 5.1. Analysis of the Filling Behavior of Semi-Solid Billet for the Thin-Wall Phone Cover

**Figure 4:**
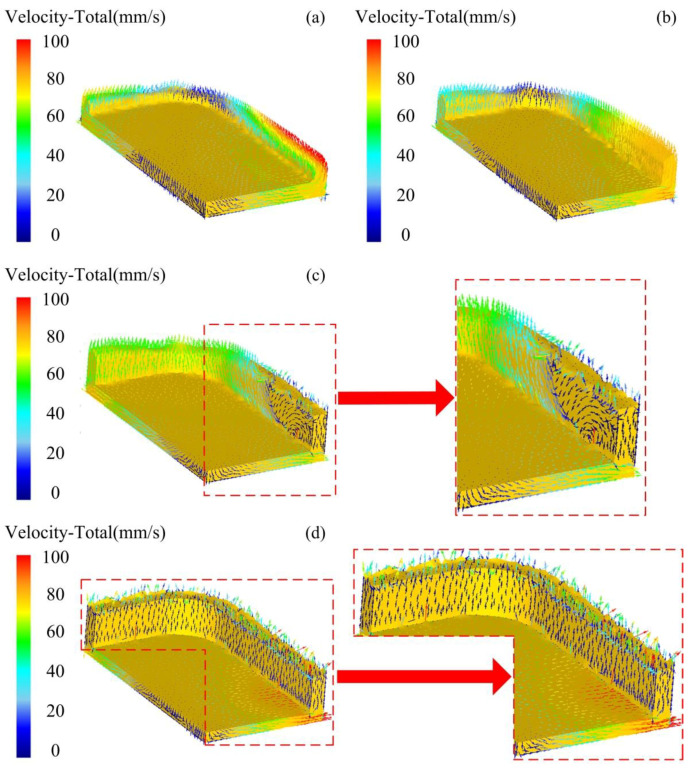
Velocity distribution during the filling process of mobile phone shell at (**a**) 0.25 mm, (**b**) 0.5 mm, (**c**) 0.75 mm, and (**d**) 1 mm displacements of the top mould.

The velocity distribution of the semi-solid billet of the mobile phone shell during the thixotropic extrusion forming process at different displacements of the top mould with the billet temperature of 630 °C, mould temperature of 400 °C, and extrusion speed of 10 mm/s is shown in Figure 4. It was found that the filling speed of the semi-solid material at the side edge was relatively high while that at the end edge was relatively low when the displacement of the top mould was 0.25 mm. This was because the semi-solid material in contact with the top mould began to flow to the side edge under the mould displacement. Therefore the upward squeeze filling effect was first observed at the side edge of the mobile phone shell due to the short material flow distance. However, the relatively few semi-solid materials were being squeezed at the end edge of the mobile phone shell due to the long flow distance. The lowest felling speed was observed at the corners of the mobile phone shell due to the relatively small amount of the semi-solid materials occupied there. 

The distribution of filling velocity at the top mould displacement of 0.5 mm is presented in Figure 4b. Similar to the displacement of 0.25 mm, the filling velocity of the semi-solid material at the end edge was lower than the side edge, which was higher than the corners. The highest velocity was located at the side edge near the symmetric line X, which was indicated by yellow vector arrows. When the displacement of the top mould was 0.75 mm, as shown in Figure 4c, the semi-solid material started to flow into the corner region of the phone shell when it flew to the side edge region forced by the top mould. The reverse flow with a low speed was observed at the side edge near the symmetric line X, which is indicated by blue vector arrows. This means that the filling of the semi-solid material at the side edge has been finished. As presented in Figure 4d, when the displacement of the top mould was 1 mm, the shape outline of the mobile phone shell is clear. The flow velocity at the side edge and corner was low, which was illustrated by the blue vector arrows. This means that the filling of the phone shell had been successfully completed at the displacement of 1 mm. 

**Figure 5:**
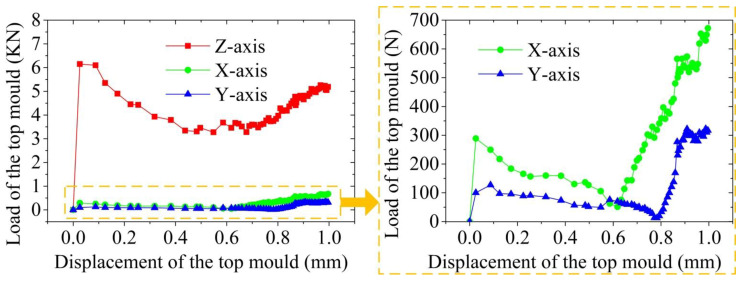
Variation of the load with the displacement of the top mould during the thixotropic extrusion process.

The load-displacement curve of the thixotropic extrusion forming under different displacements of the top mould at the billet temperature of 630 °C, die temperature of 400 °C, and the extrusion speed is 10 mm/s is shown in Figure 5. It was found that the load of the top mould at all directions first increased rapidly, then slowly reduced, and gradually rose at the end. This variation phenomenon can be explained by the thixotropic extrusion deformation mechanism of semi-solid materials. The mesh formed by solid grains was gradually broken at the beginning of extrusion during the thixotropic extrusion process of high solid fraction semi-solid materials. Therefore, plastic deformation is the major mechanism at this stage. The liquid phase can then flow out when the solid grain is deformed, so the plastic deformation of semi-solid materials included the grain sliding and partial rotation of solid grains. Consequently, the required load for the deformation was reduced under the same conditions. However, as the reaction force of the shell edges on the top mould increased during the extrusion process, the increase in mould load was observed with the further elevation in the displacement of the top mould. Although the top mould born the load from all X, Y, and Z directions, the load in the X-axis and Y-axis directions was far less than the Z-axis. Therefore, the load at Z-axis was the main load for the top mould. The displacement of the top load where the rise of the load started due to the reaction force the shell edges at X-axis was observed lower than Y-axis, which is amplified and shown in Figure 4. This was because the extrusion filling process at the side edge occurred prior to the end edge due to the unequal lengths, which caused different flow distribution at the end and side edges. Although the load at Z-axis was observed with the rising trend when the displacement of the top mould was higher than 0.5 mm, the deformation resistance was small as the billet is in the semi-solid stage. Therefore, the maximum load at the Z-axis during the thixotropic extrusion forming process was lower than 6.5 kN. 

**Figure 6:**
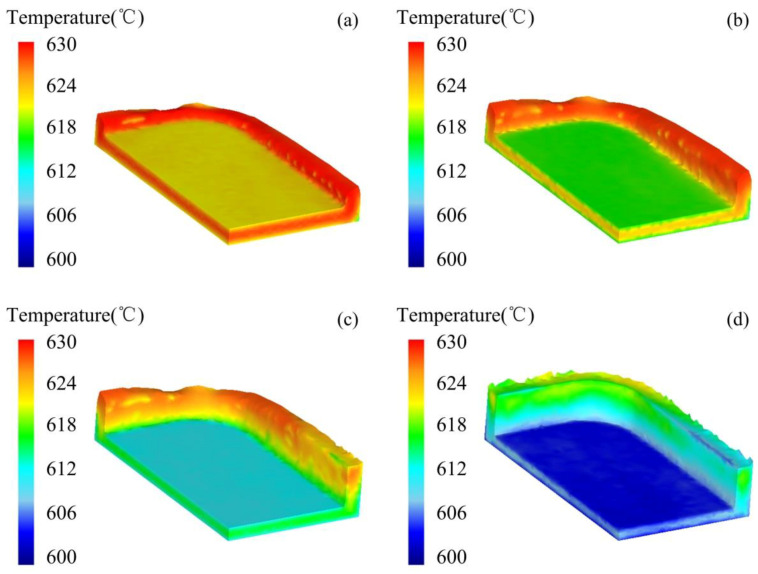
Temperature distribution during the filling process of mobile phone shell at (**a**) 0.25 mm, (**b**) 0.5 mm, (**c**) 0.75 mm, and (**d**) 1 mm displacements of the top mould.

The temperature distribution of the billet processed by the thixotropic extrusion under different displacements of the top mould at the billet temperature of 630 °C, die temperature of 400 °C, and the extrusion speed of 10 mm/s is shown in Figure 6. It can be found that the temperature at the edges was all higher than the main body of the mobile phone shell at all different displacements of the top mould. The temperature at both edges and main body reduced with the increase in the displacement. The temperature decrease in the main body was observed faster than the edges as it directly contacted the top mould during the extrusion process. When the displacement was 0.25 mm, although a decrease in the main body of the shell was observed, the temperature at edges maintained high. When the displacement was 0.5 mm, the temperature at outside surfaces of the main body contacting with the top and bottom moulds reduced to around 618 °C. At the displacement of 0.75 mm, the temperature at the outside surfaces of the main body dropped to about 612 °C, where the solidification was observed. However, the temperature at the inside space of the main body was still in the semi-solid temperature range. As the displacement of the top mould increased to 1 mm, the solidification of the main body of the mobile phone shell was finished while the temperature of the material at the end side and corner was still in the range of 612–618 °C, due to the late filling. This means that under these process parameters of the semi-solid thixotropic extrusion forming, the solidification of the main body has been finished at the end of the filling process while the temperature at the end edge and the corner were still above the solidus temperature, which is conducive to the sequential solidification of the phone shell.

### 5.2. Effects of Billet Temperature on the Solidification Behavior

**Figure 7:**
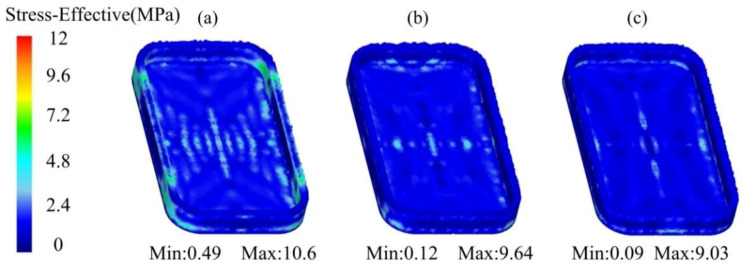
Equivalent stress distribution of the mobile phone shell at the billet temperatures of (**a**) 625 °C, (**b**) 630 °C, and (**c**) 635 °C.

The equivalent stress distribution of the mobile phone shell processed by the thixotropic extrusion at different billet temperatures under the mould temperature of 400 °C and extrusion speed of 10 mm/s is shown in Figure 7. It was found that the equivalent stress reduced with the elevation in the billet temperature. The maximum equivalent stress was 10.6, 9.64, and 9.03 MPa at the billet temperature of 625, 630, and 635 °C, respectively. The reduction in the equivalent stress was relatively large when the billet temperature increased from 625 to 630 °C. It means that increasing the billet temperature is helpful to reduce the pressure required to form the mobile phone shell during the thixotropic extrusion process. Therefore, the relatively high temperature of the blank can be designed for reducing the deformation resistance. However, the billet temperature cannot be designed too high in order to obtain the qualified microstructure and clamping strength during the semi-solid thixotropic extrusion process.

**Figure 8:**
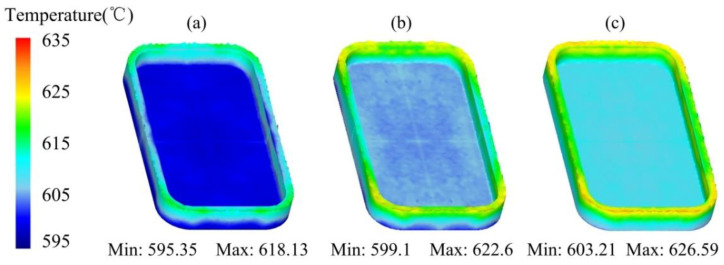
Temperature distribution of the mobile phone shell at the billet temperatures of (**a**) 625 °C, (**b**) 630 °C, and (**c**) 635 °C.

The temperature distribution of the mobile phone shell processed by the thixotropic extrusion at different billet temperatures under the die temperature of 400 °C and extrusion speed of 10 mm/s is presented in Figure 8. It can be seen that although the temperature of the phone shell elevated when the billet temperature increased during the thixotropic extrusion process, the variation of the temperature was not significant. This was mainly because, the volume of the semi-solid material was small compared to the mould, which caused a sufficient heat exchange between the mould and the semi-solid material. Consequently, no significant temperature change was observed when the thixotropic extrusion was finished. However, when the billet temperature was 625 °C, the minimum temperature of the mobile shell was found as 595.35 °C as shown in Figure 8a, which is much lower than the solidus temperature of the aluminum alloy 6063 (610 °C). This low-temperature area was observed in the side edge, which would be harmful to the successful forming of the phone shell. 

### 5.3. Effects of Mould Temperature on the Solidification Behavior

**Figure 9:**
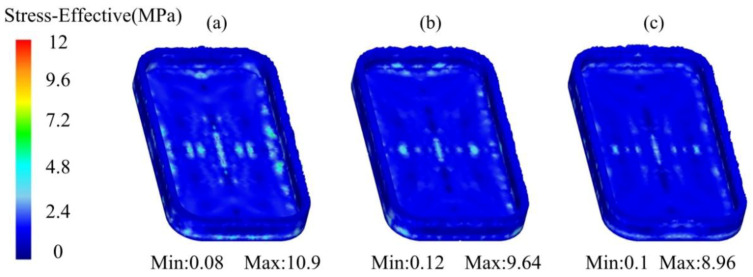
Equivalent stress distribution of the mobile shell processed by the thixotropic extrusion at the mould temperature of (**a**) 350 °C, (**b**) 400 °C, and (**c**) 450 °C.

The equivalent stress distribution of the mobile shell processed by the thixotropic extrusion at different mould temperatures under the billet temperature of 630 °C and extrusion speed of 10 mm/s is shown in Figure 9. It was found that the equivalent stress reduced with the increase in the mould temperature. The maximum equivalent stresses of the mobile phone shell parts were 10.9, 9.64 and 8.96 MPa at the mould temperatures of 350, 400 and 450 °C, respectively. The decrease in the equivalent stress from the mould temperature of 350 °C to 400 °C was observed more significant than that from the mould temperature of 400 °C to 450 °C. This means that increasing the mould temperature is able to reduce the pressure required for forming during the thixotropic extrusion molding process. 

**Figure 10:**
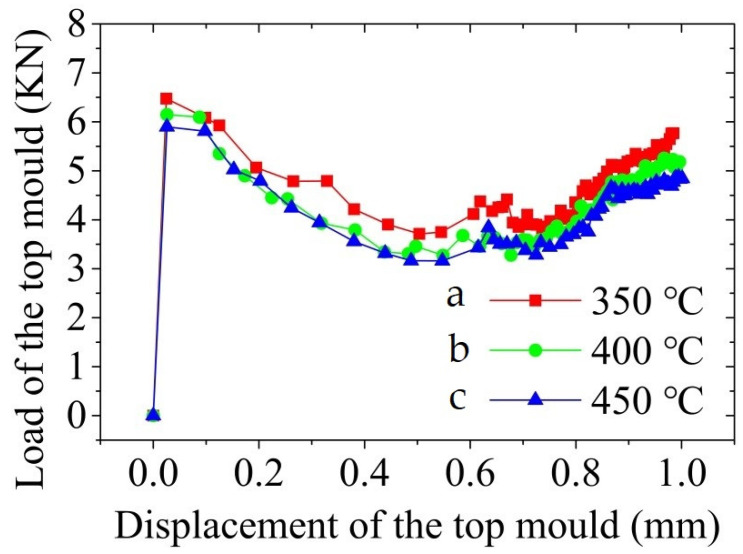
Load-displacement curve of the top mould during the thixotropic extrusion at the mould temperature of (**a**) 350 °C, (**b**) 400 °C, and (**c**) 450 °C.

Figure 10 shows the load-displacement curve of the top mould during the thixotropic extrusion at different mould temperatures under the billet temperature of 630 °C and extrusion speed of 10 mm/s. It was found that the load of the top mould reduced with the elevation in the mould temperature. The load of the top mould was obtained as the maximum value in the load-displacement curve, which was found at a displacement of less than 0.1 mm. From Figure 10, it can be observed that the load of the top moulds are 6.5, 6.1, and 5.9 kN at the mould temperature of 350, 400, and 450 °C, respectively.

**Figure 11:**
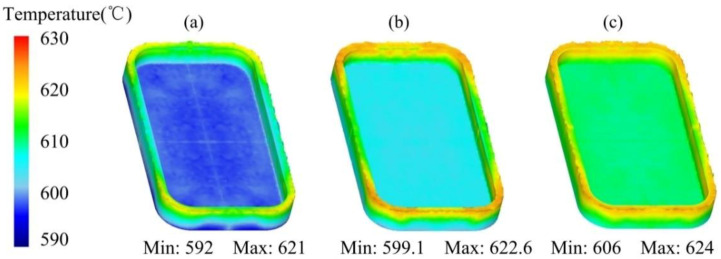
Temperature distribution of the mobile phone shell at the mould temperatures of (**a**) 350, (**b**) 400, and (**c**) 450 °C.

Figure 11 shows the temperature distribution of the mobile phone shell processed by the thixotropic extrusion at different mould temperatures under the billet temperature of 630 °C and extrusion speed of 10 mm/s. It can be found that the general temperature of the phone shell elevated with the enhancement in the mould temperature. The temperature of the semi-solid material at the edges was higher than the main body in all mould temperature cases. As shown in Figure 11a, most of the side edge temperature of the phone shell was below 615 °C when the mould temperature was 350 °C, which may lead to a high-temperature solid forming rather than the semi-solid forming. Therefore, the mould temperature is recommended as 400 °C or higher for a successful semi-solid forming of the mobile phone shell.

### 5.4. Effects of Extrusion Speed on the Solidification Behavior

**Figure 12:**
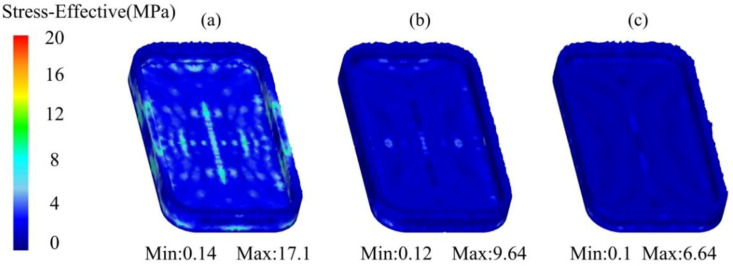
Equivalent stress distribution of the mobile phone shell processed by the thixotropic extrusion at the extrusion speed of (**a**) 5 mm/s, (**b**) 10 mm/s, and (**c**) 15 mm/s.

The equivalent stress distribution of the mobile phone shell processed by the thixotropic extrusion at different extrusion speeds under the billet temperature of 630 °C and a mould temperature of 400 °C is shown in Figure 12. It was found that the equivalent stress decreased with the elevation in the extrusion speed. The maximum equivalent stresses were 17.1, 9.64, and 6.64 MPa at the extrusion speed of 5, 10, and 15 mm/s, respectively when the forming was finished. The reduction in the equivalent stress when the extrusion speed increased from 5 to 10 mm/s was larger than that from 10 to 15 mm/s. Therefore, the required pressure for forming the mobile phone shell can be reduced by increasing the strain rate, but a high strain rate also causes a high requirement on the performance of the forming equipment, so the design of the extrusion speed is determined by the forming equipment performance. It was also found that the most uniform distribution of the equivalent stress was observed at the extrusion speed of 15 mm/s. This means that the deformation resistance of the material was the lowest at the extrusion speed of 15 mm/s, which was beneficial to the material filling. 

**Figure 13:**
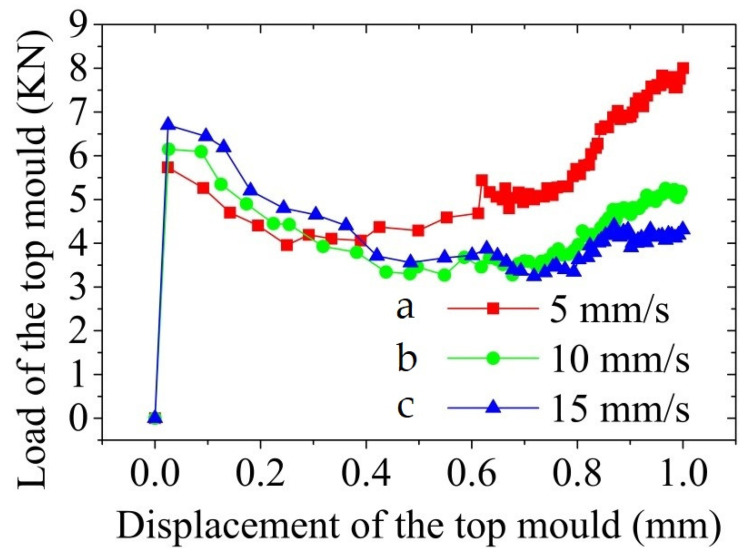
Top mould load during the thixotropic extrusion at the extrusion speed of (**a**) 5 mm/s, (**b**) 10 mm/s, and (**c**) 15 mm/s.

Figure 13 shows the load-displacement curve of the top mould at Z-axis during the thixotropic extrusion at different extrusion speeds under the billet temperature of 630 °C and mould temperature of 400 °C. It can be seen that the initial maximum load on the top mould increased when the extrusion speed rose in the semi-solid thixotropic extrusion forming process of the mobile phone shell, which means that requirements for the tonnage of the forming equipment were high at a high extrusion speed. However, when the displacement of the top mould reached 0.3 mm, the load of the top mould at the extrusion speed of 5 mm/s surpasses that at the extrusion speed of 10 mm/s, which further exceeded the load at the extrusion speed of 15 mm/s when the displacement increased to 0.4 mm. With the further growth in the displacement of the top mould above 0.7 mm/s, the maximum load of the top mould was observed at the extrusion speed of 5 mm/s while the minimum value was found at the extrusion speed of 15 mm/s. 

The phenomenon mentioned above in Figure 13 was because, with the increase in the top mould speed, part of the solid phase grain agglomerates in the semi-solid microstructure were destroyed by the stronger shearing effect, which promoted the release of the liquid phase wrapped by the agglomerates and increased liquid content inside the semi-solid material. Therefore, the filling process of the semi-solid material becomes easier at a higher extrusion speed due to the easier slid and rotation of the solid grains. Furthermore, there was a relatively large temperature difference between the billet temperature and the mold temperature during the semi-solid thixotropic extrusion process. The percentage of the semi-solid material that solidified due to the cooling of the mould reduced with the increase in the top mould speed, which was beneficial for the filling process.

It was also found that the load of the top mould at the Z-axis first reduced with the elevation in the displacement and then increased when the displacement further augmented at all three extrusion speeds. At the displacement of 1.0 mm, the load of the top mould increased to 5.8 kN at the extrusion speed of 5 mm/s while that elevated to 6.1 and 6.8 kN when the extrusion speeds were 10 and 15 mm/s, respectively. However, the turning points of the variation were different at different extrusion speeds. The increasing trend started at the displacement of 0.7 mm under the extrusion speed of 10 mm/s and 15 mm/s while that took place at the displacement of 0.4 mm when the extrusion speed was 5 mm/s. This means that under the same other operating conditions when the displacement of the top mould reached 0.4 mm at the extrusion speed of 5 mm/s, the cooling and solidification took place during the filling process, which led to the increase in the top mould load.

**Figure 14:**
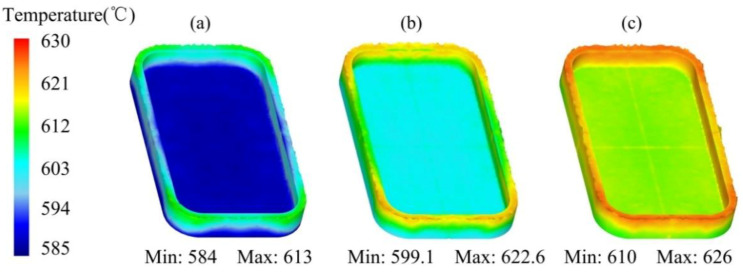
Temperature distribution of the mobile phone shell processed by the thixotropic extrusion at the extrusion speed of (**a**) 5 mm/s, (**b**) 10 mm/s, and (**c**) 15 mm/s.

The temperature distribution of the mobile phone shell processed by the thixotropic extrusion at different extrusion speeds under the billet temperature of 630 °C and a mould temperature of 400 °C is shown in Figure 14. It can be found that the general temperature of the mobile phone shell is augmented with the elevation in the extrusion speed. As shown in Figure 14a, at the extrusion speed of 5 mm/s, the highest temperature of the phone shell was observed as 613 °C, which was lower than the solidus temperature (i.e., 615 °C). This means that the forming process of the mobile phone shell was turned into high-temperature solid forming instead of semi-solid forming. It is shown in Figure 14b,c that the maximum temperature of the phone shell was 622.6 °C and 626 °C, respectively. This means that an appropriate elevation in the extrusion speed of the top mould can keep the temperature of the phone shell in the semi-solid temperature range so that the entire thixotropic extrusion process can be successfully completed in the semi-solid range. Based on the simulation results in this study, the extrusion speed is recommended as 10 mm/s or higher for realizing the filling process of the material at the semi-solid state. 

### 5.5. Optimal Process Parameters for the Semi-Solid Thixoforming of the Thin-Wall Phone Cover

According to the analysis of Figure 4, Figure 5, Figure 6, Figure 7, Figure 8, Figure 9, Figure 10, Figure 11, Figure 12, Figure 13 and Figure 14, the effects of the billet temperature, mould temperature, and extrusion speed of the top mould on the forming of mobile phone shells in the semi-solid thixotropic extrusion forming process can be summarized as follows:

(a) The equivalent stress of the blank decreased while the forming temperature augmented with the elevation in the billet temperature. Therefore, the elevation in the billet temperature was able to reduce the pressure required in the thixotropic extrusion forming process of the mobile phone shell. However, the growth in the billet temperature would inevitably cause the coarsening and growth of the semi-solid microstructure during the semi-solid thixotropic extrusion process. Hence, both the forming pressure and the semi-solid microstructure quality need to be considered in the determination of the billet temperature. According to the simulation results, when the billet temperature was 625 °C, the minimum temperature of the mobile shell was found as 595.35 °C as shown in Figure 8a, which is much lower than the solidus temperature of the aluminum alloy 6063 (610 °C). This low-temperature area was observed in the side edge, which would be harmful to the successful forming of the phone shell. Therefore, the billet temperature is recommended as 630 °C or higher. 

(b) The equivalent stress and the load on the top mould of the mobile phone shell decreased with the elevation in the mould temperature, which means that the requirements for the tonnage of the forming equipment reduced when the mould temperature augmented. According to the numerical simulation results, when the mold temperature was 350 °C, the temperature of the material at most of the shell edges was observed below 615 °C. This may lead to high-temperature solid-state forming instead of semi-solid forming. Therefore, to successfully realize the forming of mobile phone shell parts in a semi-solid state, the mold temperature is recommended to be 400 °C or higher.

(c) The equivalent stress in the blank reduced when the extrusion speed of the top mould elevated. A significant decrease in the equivalent stress was observed when the extrusion speed was augmented from 5 to 10 mm/s, which helped to reduce the pressure required to form the mobile phone shell, but it caused higher requirements on the performance of the forming equipment. Furthermore, the temperature of the phone shell increased when the extrusion speed of the top mould rose, which helped to complete the forming process of the entire shell at the temperature within the semi-solid range. According to the simulation results, when the extrusion speed was 5 mm/s, the maximum temperature of the phone shell during the forming process was 613 °C, which was lower than the solidus temperature of 615 °C. This caused a high-temperature solid forming of the mobile phone shell instead of the semi-solid forming. Therefore, the extrusion speed of the top mould is recommended as 10 mm/s or higher. 

The effects of process parameters on the SSTEF of phone shells obtained from all simulations can be found in Table 3. In summary, to save energy and reduce the production cost, the optimal process parameters for semi-solid thixotropic extrusion forming of mobile phone shell parts are the billet temperature of 630 °C, the mould temperature of 400 °C, and the extrusion speed of 10 mm/s. 

**Figure 15:**
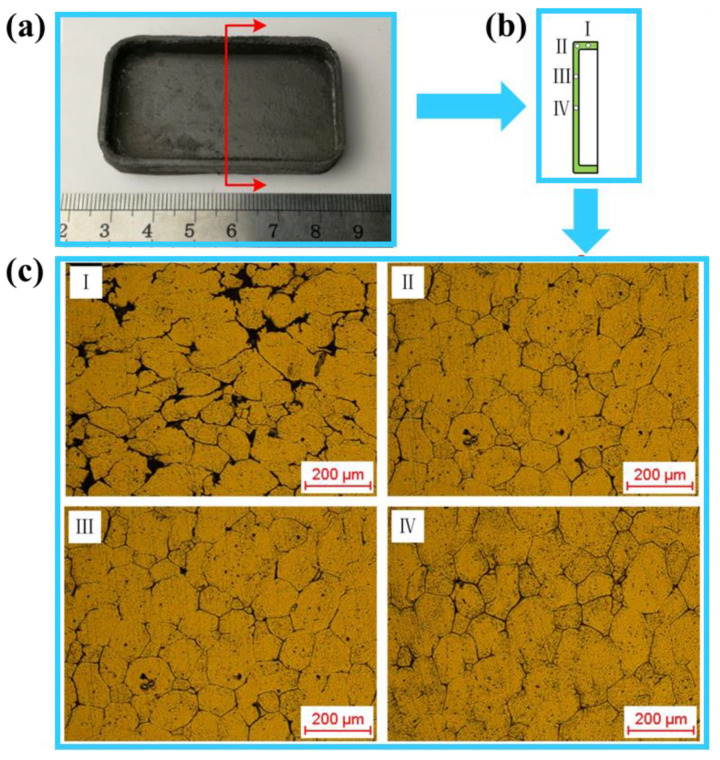
The macromorphology and microstructure at different sampling positions of the mobile phone shell process by semi-solid thixotropic extrusion forming: (**a**) macro morphology, (**b**) sampling positions, and (**c**) microstructure.

The macromorphology and the microstructure at different sampling locations of the aluminum alloy 6063 mobile phone shell processed by the semi-solid thixotropic extrusion at the billet temperature of 630 °C, mould temperature of 400 °C, and the extrusion speed of 10 mm/s are shown in Figure 15. It can be seen from Figure 15a that the mobile phone shell was fully filled with clear outlines and smooth surfaces. There were no obvious macro defects such as cracks, mold sticking, entrainment, shrinkage, etc., indicating that qualified and relatively stable filling process of semi-solid materials under these optimal process parameters. 

Figure 15c shows that although the solid grains at Position I of the phone shell were observed spherical, more obvious local liquid segregations were revealed in the microstructure compared to Positions II, III, and IV. The liquid phase content was more concentrated at the edges in comparison to the main body. This was mainly because the liquid phase was easy to flow to the edges of the phone shell in the initial stage of forming due to the excellent fluidity. It was also found that excellent semi-solid microstructure characteristics were observed at all sampling positions in the main body of the phone shell (Positions II, III, and IV).

These microstructure characteristics can be summarized as: (1) the solid grains were all small and nearly spherical; (2) the uniformity degree of the microstructure was relatively high, and the variation in the grain size was small; (3) no obvious solid-liquid separation was observed in the microstructure. Therefore, the microscopic morphology of the mobile phone shell can be maintained almost the same as the semi-solid material before the forming process.

To sum up, in the filling process of semi-solid thixotropic extrusion, the flow of the semi-solid material was mainly in the state of liquid phase wrapping the spherical solid grains. However, at the edge position of the filling product, the local liquid phase segregation would take place due to the prior flow of the liquid phase. Moreover, the rotation and sliding of solid grains are also accompanied by the filling process. Less liquid phase segregation was observed in the packing stage after the filling process as the liquid phase that wrapped the solid grains was solidified under the pressure. 

The average grain size and the shape factor of solid grain at four different positions in Figure 15 are shown in Table 4. The solid grains in the microstructure were small, uniform, and nearly spherical. The average grain size varied from 73 to 79 μm. in these four positions while the shape factor changed from 0.74–0.77. 

The lowest grain size was found at Position I while the largest shape factor was observed in Position III. The highest average grain size and the lowest shape factor were found at Positions IV and I, respectively. Therefore, the average grain size of the microstructure for the whole product was then obtained as 76.92 μm and the shape factor was found as 0.76.

## 6. Conclusions

(1) The semi-solid materials contacting the top mould filled the edges prior to the main body of the aluminum alloy 6063 mobile phone shell during the semi-solid thixotropic extrusion forming process. The equivalent strain of the material contacting the top mould elevated with the augment in the displacement of the top mould. The load of the top mould in different directions first grew rapidly, then slowly reduced, and finally slightly increased when the displacement of the top mould enlarged. The temperature of solid material declined with the elevation in the displacement of the top mould. The fastest reduction in the temperature was observed in the region in contact with the top mould.

(2) The equivalent stress and the top mould load descended with the elevation in the billet temperature, which caused a lower tonnage requirement of the forming equipment. The general temperature of the phone shell augmented when the billet temperature increased, which was beneficial to the forming process completed at the temperature within the semi-solid range. The equivalent stress of the phone shell at the extrusion speed of 10 mm/s was more uniform compared to 5 mm/s, so the filling process can be conducted more smoothly at the extrusion speed of 10 mm/s. The equivalent stress of the phone shell at the extrusion speed of 10 mm/s was also found lower than 5 mm/s, which can guarantee the filling process is performed with the material at the semi-solid state.

(3) According to the analysis of the simulation results, the optimal process parameters for preparing aluminum alloy 6063 mobile phone shell by semi-solid thixotropic extrusion process were the billet temperature of 630 °C, the mould temperature of 400 °C, and the top mould speed of 10 mm/s. It was found from the experimental result that under these optimal parameters, the finished mobile phone shell was fully filled with a clear outline and a smooth surface, where the solid grains in the microstructure were small, uniform, and nearly spherical. No obvious solid-liquid separation was observed in the microstructure of the final product.

## Figures and Tables

**Table 1:** Chemical composition of the 6063 aluminium alloy (wt.%).

Mg	Si	Mn	Zn	Fe	Cu	Ti	Cr	Al
0.51	0.39	0.03	0.02	0.15	0.01	<0.001	<0.005	Balance

**Table 2:** Process parameters for the SSTEF in the simulation.

Group No.	Billet Temperature (°C)	Mould Temperature (°C)	Extrusion Speed (mm/s)
I	630	400	10
II	625, 630, 635	400	10
III	630	350, 400, 450	10
IV	630	400	5, 10, 15

**Table 3:** Effects of process parameters on the SSTEF of mobile phone shells.

Process Parameters	Results	Recommended Process Parameters
Billet temperature	➢ The equivalent stress of the blank decreased while the forming temperature augmented with the elevation in the billet temperature; ➢ When the billet temperature was 625 °C, the minimum temperature of the mobile shell was found as 595.35 °C, which is much lower than the solidus temperature of the aluminum alloy 6063 (610 °C).	630 °C or higher
Mould temperature	➢ The equivalent stress and the load on the top mould of the mobile phone shell decreased with the elevation in the mould temperature; ➢ When the mold temperature was 350 °C, the temperature of the material at most of the shell edges was observed below 615 °C.	400 °C or higher
Extrusion speed	➢ The equivalent stress in the blank reduced when the extrusion speed of the top mould elevated, and a significant decrease in the equivalent stress was observed when the extrusion speed was augmented from 5 to 10 mm/s; ➢ When the extrusion speed was 5 mm/s, the maximum temperature of the phone shell during the forming process was 613 °C, which was lower than the solidus temperature of 615 °C.	10 mm/s or higher

**Table 4:** Average grain size and shape factor of solid grain at the four different positions.

Positions	Average Grain Size (μm)	Shape Factor
Position I	73	0.74
Position II	76.6	0.76
Position III	78.37	0.77
Position IV	79.69	0.76
Average of Position I, II, III, and IV	76.92	0.76

## Data Availability

The data presented in this study are available on request from the corresponding author. The data are not publicly available due to project confidentiality requirements.

## References

[1] Bayramoglu M., Polat H., Geren N. (2008). Cost and performance evaluation of different surface treated dies for hot forging process. J. Mater. Process. Technol..

[2] Guo Y., Wang Y., Zhao S. (2020). Experimental Investigation and Optimization of the Semisolid Multicavity Squeeze Casting Process for Wrought Aluminum Alloy Scroll. Materials.

[3] Agarwal M., Srivastava R. (2016). Influence of Solid Fraction Casting on Microstructure of Aluminum Alloy 6061. Mater. Manuf. Process..

[4] Pola A., Tocci M., Kapranos P. (2018). Microstructure and Properties of Semi-Solid Aluminum Alloys: A Literature Review. Metals.

[5] Ragab K.A., Bouazara M., Chen X.-G. (2019). Quality Index Charts of Al-Si-Mg Semi Solid Alloys Subjected to Multiple Temperatures Aging Treatments and Different Quenching Media. Materials.

[6] Wang Y., Zhao S., Zhang C. (2018). Microstructures and mechanical properties of semi-solid squeeze casting ZL104 connecting rod. Trans. Nonferr. Met. Soc. China.

[7] Jiang J.-F., Wang Y., Du Z.-M., Luo S.-J. (2012). Microstructure and properties of AZ80 alloy semisolid billets fabricated by new strain induced melt activated method. Trans. Nonferr. Met. Soc..

[8] Xu Y., Chen C., Jia J., Zhang X., Dai H., Yang Y. (2018). Constitutive behavior of a SIMA processed magnesium alloy by employing repetitive upsetting-extrusion (RUE). J. Alloys Compd..

[9] Wang Y., Zhao S., Zhao X., Zhao Y. (2017). Microstructural coarsening of 6061 aluminum alloy semi-solid billets prepared via recrystallization and partial melting. J. Mech. Sci. Technol..

[10] Wang Y., Zhao S., Zhang C. (2017). Microstructural evolution of semisolid 6063 aluminum alloy prepared by recrystallization and partial melting process. J. Mater. Eng. Perform..

[11] Haghayeghi R., Zoqui E.J., Halvaee A., Emamy M. (2005). An investigation on semi-solid Al-7Si-0.3Mg alloy produced by mechanical stirring. J. Mater. Process. Technol..

[12] Van Thuong N., Zuhailawati H., Seman A.A., Huy T.D., Dhindaw B.K. (2015). Effects of Processing Parameters on Microstructure Evolution of Al-7Si-Mg Alloy by Cooling Slope Casting. J. Mater. Eng. Perform..

[13] Zhang L., Guohua W.U., Wang S., Ding W. (2015). Effect of cooling condition on microstructure of semi-solid AZ91 slurry produced via ultrasonic vibration process—TNMSC. China Foundry.

[14] Fu J.L., Jiang H.J., Wang K.K. (2018). Influence of Processing Parameters on Microstructural Evolution and Tensile Properties for 7075 Al Alloy Prepared by an ECAP-Based SIMA Process. Acta Metall. Sin..

[15] Wang Y., Zhao S., Zhao X., Zhao Y. (2017). Effects of isothermal treatment parameters on the microstructure of semisolid alloys. Mater. Sci. Technol..

[16] Jiang J.F., Wang Y., Luo S.J. (2007). Application of equal channel angular extrusion to semi-solid processing of magnesium alloy. Mater. Charact..

[17] Chen Q., Zhao Z.D., Chen G., Wang B. (2015). Effect of accumulative plastic deformation on generation of spheroidal structure, thixoformability and mechanical properties of large-size AM60 magnesium alloy. J. Alloys Compd..

[18] Wang Y., Zhao S., Guo Y., Liu K., Zheng S. (2021). Deformation Characteristics and Constitutive Equations for the Semi-Solid Isothermal Compression of Cold Radial Forged 6063 Aluminium Alloy. Materials.

[19] Wang Y., Zhao S., Zhang C. (2017). Grain Refinement of Aluminum Alloy Bar by a Modified RAP Process for Semi-Solid Forming. Mater. Trans..

[20] Wang Y., Zhao S., Zhang C. (2016). Application of Radial Forging and Remelting Treatment to Prepare Semi-Solid Billet of AlMg0.7Si Alloy. Solid State Phenom..

[21] Chen G., Zhang S., Zhang H., Han F., Wang G., Chen Q., Zhao Z. (2018). Controlling liquid segregation of semi-solid AZ80 magnesium alloy by back pressure thixoextruding. J. Mater. Process. Technol..

[22] Luo M., Li D., Midson S.P., Qu W., Zhu Q., Fan J. (2019). Model for Predicting Radial Temperature Distribution of Semi-Solid Slug Produced by Swirled Enthalpy Equilibration Device (SEED) Process. J. Mater. Process. Technol..

